# Label-Free Surface-Enhanced Raman Scattering for Genomic DNA Cytosine Methylation Reading

**DOI:** 10.3390/molecules30020403

**Published:** 2025-01-18

**Authors:** Kazi Morshed Alom, Anastasiia Tukova, Nana Lyu, Alison Rodger, Yuling Wang

**Affiliations:** 1 School of Natural Sciences, Macquarie University, Sydney, NSW 2109, Australia; kazimorshed.alom@hdr.mq.edu.au (K.M.A.); anastasiia.tukova@hdr.mq.edu.au (A.T.); nana.lyu@mq.edu.au (N.L.); 2 Research School of Chemistry, The Australian National University, Canberra, ACT 2601, Australia

**Keywords:** DNA methylation, CpG site, gold nanoparticles, label-free surface-enhanced Raman scattering (SERS)

## Abstract

DNA methylation has been widely studied with the goal of correlating the genome profiles of various diseases with epigenetic mechanisms. Multiple approaches have been developed that employ extensive steps, such as bisulfite treatments, polymerase chain reactions (PCR), restriction digestion, sequencing, mass analysis, etc., to identify DNA methylation. In this article, we report a facile label-free surface-enhanced Raman scattering (SERS) spectroscopy system that utilizes gold nanoparticles (AuNPs) for signal enhancement of methylated DNA. The key innovation of this work is to use anionic nanoparticles at a high ionic strength to introduce the aggregation of AuNPs with anionic DNA. When target methylated DNA is present, the presence of a methyl group in the cytosine C5 position of CpG sites induces a Raman peak at 1350 cm^−1^. Our amplification-free system has a limit of detection (LOD) of 5% and a limit of quantification (LOQ) of 16% with good specificity. The method was applied to determine the hypermethylated levels of the germline of colorectal cancer cell lines SW48 and SW480. Our simple label-free method holds the potential to read the disease-associated methylation of genomic DNA.

## 1. Introduction

Among various types of epigenetic modifications, DNA methylation has been identified as critical due to its association with disease pathways [1,2]. It commonly refers to the addition of an extra methyl (–CH_3_) group to C5 of the cytosine (C) base in CpG dinucleotides, regions of DNA where a cytosine nucleotide is followed by a guanine nucleotide [3,4]. Cytosine methylation plays a crucial role in gene expression during genomic imprinting, cellular differentiation, and in diseases through gene silencing [5,6]. Particularly, promoter sequence hypermethylation is reported to exhibit silencing effects [7,8].

While gene-specific methylation has biological roles, aberrant methylation throughout the genome has downstream impacts on organisms. Approximately 70% of CpG dinucleotides in the human genome are reported to be methylated [9,10,11]. In many cases, these methylated cytosines are then deaminated to thymine and are not repaired by the DNA repair mechanism, contributing to irreversible mutation in descendent cells [9]. Cytosine methylation also contributes to chromosomal instability in embryonic and germline developmental pathways [12,13,14,15]. These associations make global DNA methylation a significant biomarker.

Bisulfite-treated polymerase chain reaction (PCR) [16,17,18,19], high-performance liquid chromatography (HPLC) [20,21,22,23], and enzyme-linked immunosorbent assay (ELISA) [24,25] are the gold standard methods used for global DNA methylation detection. However, following the bisulfite conversion, the absence of all cytosine bases affects DNA hybridization, and thus PCR accuracy. HPLC can be less sensitive and requires a higher amount of sample than is needed for clinical applications, and non-specific antibody binding can reduce the selectivity of ELISA.

Recently, surface-enhanced Raman scattering (SERS) spectroscopy has gained attention regarding biosensing applications. It utilizes the basic principles of Raman spectroscopy with enhancement of the inelastic scattering of photons by molecules adsorbed onto the surfaces of plasmonic structures (usually gold or silver nanostructures) [26]. Multiple label-free systems utilizing plasmonic nanoparticles and aggregation-based SERS enhancement for global DNA methylation have been reported [27,28,29,30]. Some of these systems produce impressive detection limits, but with tedious processes. So, we aimed to demonstrate a simple global DNA methylation detection procedure utilizing gold nanoparticles (AuNPs) for SERS enhancement.

**Figure 1:**
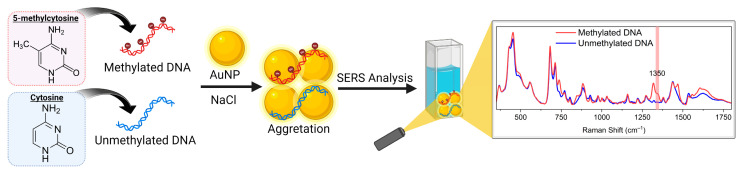
Schematic representation of label-free AuNPs aggregation-mediated SERS method used for global DNA methylation reading. NaCl-induced aggregation of AuNPs adsorbed DNA onto its surface and allowed differentiation of characteristic SERS peaks of both methylated and unmethylated DNA. Figure created in BioRender.com.

The goal of this work was to develop a simple one-pot label-free SERS method that would allow us to read the presence of methylation in the target DNA without any preprocessing with sodium bisulfite or amplification via PCR. We simply mixed our target anionic DNA with anionic AuNPs and sodium chloride, which created DNA–AuNP assemblies. AuNPs aggregation positioned DNA targets in SERS hot spots (between nanoparticles) and produced a strong Raman signal following laser excitation. Particularly, a distinct 1350 cm^−1^ peak was observed for synthetic methylated targets (Figure 1). Finally, analysis of colorectal cancer genomic DNA generated methylation-specific peaks, which further affirmed the potential of our approach.

## 2. Results and Discussion

### 2.1. Characterization of Nanoparticles Used for SERS Enhancement

**Figure 2:**
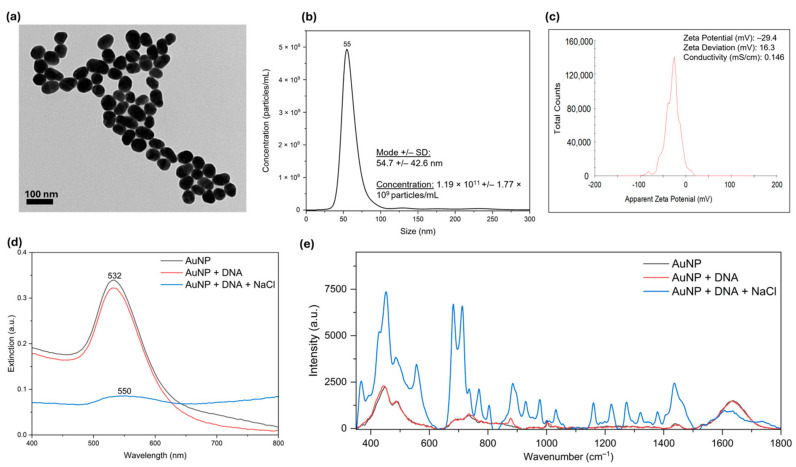
(**a**) Transmission electron microscopy (TEM) image showing spherical shape of AuNPs; (**b**) NTA showing size distribution and concentration of AuNPs; (**c**) zeta potential distribution revealing surface charge of AuNPs; (**d**) extinction spectra of AuNPs, AuNPs with DNA, and AuNPs with DNA and NaCl; and (**e**) SERS spectra of AuNPs, DNA, and aggregation state with NaCl.

We chose to use spherical gold nanoparticles (AuNPs) with a large surface/volume ratio, as well as a reproducible size and stability [31,32,33,34]. AuNPs with a size of 55 nm were adopted for our SERS analysis due to their high localized surface plasmon resonance (LSPR) properties, signal enhancement of over 100-fold, and high scattering/absorption ratio [35,36,37,38,39,40,41]. We synthesized citrate-capped AuNPs following the Turkevich method [42] and confirmed their shape and approximate size by transmission electron microscopy (TEM) (Figure 2a). The size and concentration of AuNPs were evaluated with nanoparticle tracking analysis (NTA). As indicated in Figure 2b, the average hydrodynamic size of AuNPs was 55 nm with a concentration of 1.19 × 10^11^ particles/mL. Citrate-capping results in negatively charged particles and zeta potential measurements showed a −29.4 mV surface charge in our particles (Figure 2c). Further, extinction spectra showed a characteristic peak at 532 nm for our AuNPs, which remained the same in the presence of DNA at low ionic strength but decreased and shifted to 550 nm on the addition of 0.05 mM NaCl (final concentration), indicating salt-induced aggregation of DNA and AuNPs (Figure 2d). As both AuNPs and DNA were negatively charged, no DNA SERS signal was observed when they were mixed, as the DNA could not bind to the SERS hotspot. However, the addition of NaCl induced their interaction and produced a strong SERS signal from the DNA (Figure 2e).

### 2.2. Identification of Spectra Differences Between Cytosine and 5-Methylcytosine Bases

**Figure 3:**
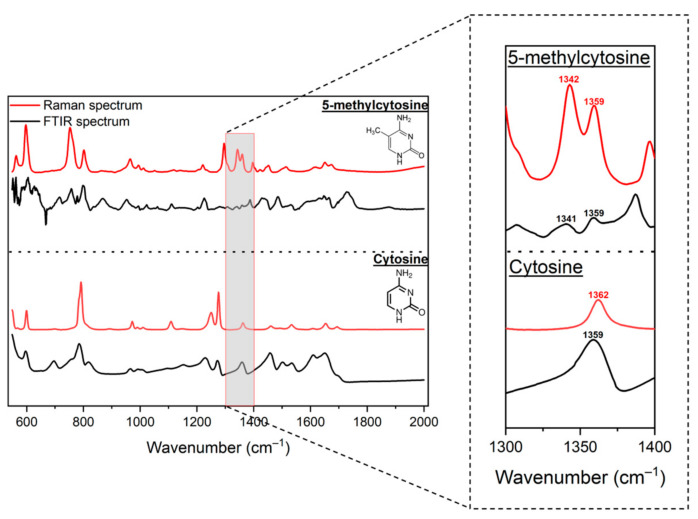
FTIR absorbance and Raman spectra of cytosine and 5-methylcytosine molecules in solid state revealing differences in peak positions; 5-methylcytosine had a specific peak at 1342 cm^−1^.

Reported studies of AuNP-based SERS analysis have shown 5-methylcytosine (5mC) to have Raman frequency changes near 785, 1000, and 1350 cm^−1^ due to the –CH_3_ group’s vibrational modes [43,44,45,46]. To identify target peaks for 5mC versus C for this work, we measured the normal Raman and Fourier transform infrared (FTIR) absorption spectra of the solid samples (Figure 3). We found similarities between the normal Raman and FTIR spectra among the same molecules. However, two distinct regions showed the largest dissimilarities between these two molecules. Notably, the peak at 791 cm^−1^ for C was replaced with 752 cm^−1^, and 802 cm^−1^ peaks for 5mC. In addition, 5mC had two peaks at 1342 cm^−1^ and 1359 cm^−1^, whereas C only had one peak at 1359 cm^−1^.

**Figure 4:**
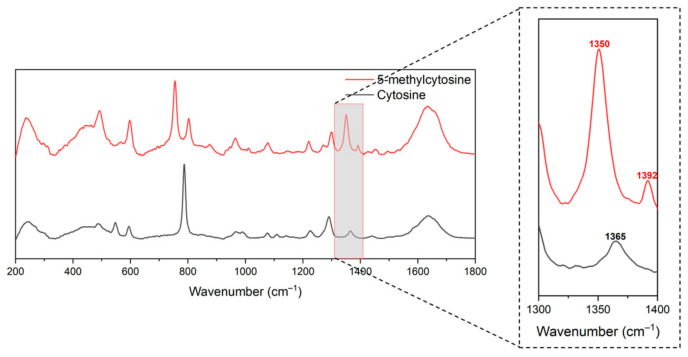
Raman spectra of cytosine and 5-methylcytosine dissolved in PBS in quartz cuvette for further confirmation of peak differences between these molecules.

From there, we moved on to measure the Raman spectra of these two molecules in dissolved condition (as this would resemble DNA samples in further steps). We dissolved as much C and 5mC in deionized water as possible and then filtered the solutions to remove undissolved material. We weighed the undissolved material and determined the solution concentrations for C (63 mM) and 5mC (77 mM). We measured Raman spectra of C (final concentration 5.25 mM) and 5mC (final concentration 6.41 mM) in 1× phosphate buffer saline (PBS). A peak specific to 5mC occurred at 1350 cm^−1^ (Figure 4). Markedly, the 1342 and 1359 cm^−1^ peaks of the solid 5mC sample in Figure 3 merged into this one peak for the same sample suspended in PBS, as shown in Figure 4. Peaks in this region have been reported to be associated with CpG methylation by many groups. For further investigation of the C5 methylation of our DNA samples, we chose to compare differences around this specific peak position [47,48,49].

### 2.3. Quantification of DNA Methylation with AuNP Aggregation-Mediated SERS

From the cytosine molecule level, we moved on to evaluate methylation status in DNA strands. We chose a high CpG containing short DNA and treated it with CpG methyltransferase (M.SssI) to make the control methylated DNA, and used the untreated one as the control unmethylated DNA (Table 1). Based on our previous experience of DNA precipitation with highly charged cations, such as polyamines and metal complexes [50,51,52], we tried anionic particles and anionic DNA with the assembly controlled by ionic strength. In order to position the DNA in the SERS hotspot, we mixed the DNA with AuNPs and aggregated them by adding high levels of NaCl and PBS (which also contains NaCl). The salt was added to reduce charge–charge repulsion and allow the aggregation of AuNPs with DNA without forming a precipitate at the bottom.

**Figure 5:**
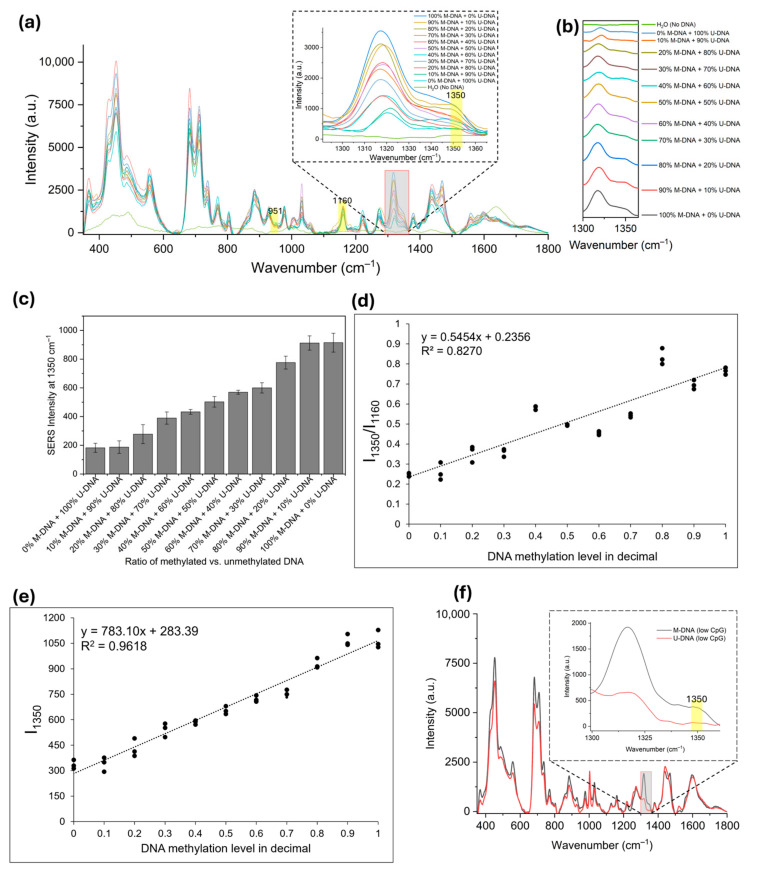
(**a**) SERS spectra of percent-wise methylated and unmethylated (total 250 ng) control DNA showing peak intensity difference at 1350 cm^−1^; (**b**) stacked SERS spectra near 1350 cm^−1^ region showing gradual decrease in intensity with reducing methylation in DNA; (**c**) bar graph representing minimum level of detectable DNA methylation in comparison to unmethylated DNA; (**d**) linear regression graph based on intensity ratios of 1350 and 1160 cm^−1^; (**e**) linear regression graph based on intensity at 1350 cm^−1^; and (**f**) SERS spectra of methylated and unmethylated DNA with lower CpG density. All samples contained a total of 250 ng of DNA. Error bars represent standard deviations of three different samples.

**Figure 6:**
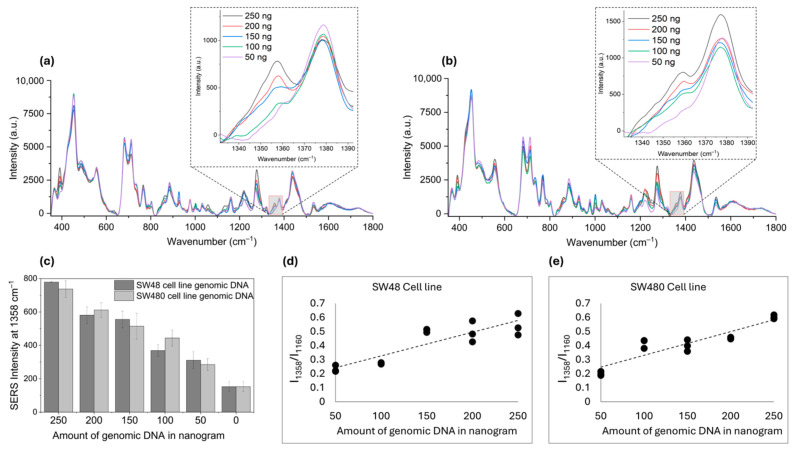
(**a**) SW48 cell line and (**b**) SW480 cell line genomic DNA SERS spectra showing characteristic methylation peak at 1358 cm^−1^; (**c**) bar graph representing decrease in SERS intensity with decreasing amount of genomic DNA of two cell lines; and (**d**,**e**) linear regression graphs for SW48 and SW480 cell lines, respectively, based on their concentrations against their intensity ratios of 1358 and 1160 cm^−1^. Error bars represent standard deviation of three different samples.

These spectra were recorded after mixing methylated and unmethylated DNA in a percent-wise ratio (Figure 5a). For methylated DNA, there was a peak present at 1350 cm^−1^. As the ratio of methylated DNA decreased, the intensity at 1350 cm^−1^ decreased proportionately, and for 100% unmethylated DNA the 1350 cm^−1^ peak plateaued near the H_2_O level (Figure 5b). The bar diagram generated using the information in Figure 5a allowed us to assign a specific methylation percentage to a certain intensity value (Figure 5c). To construct a linear regression graph, we identified 951 (related to adenosine) and 1160 cm^−1^ (related to deoxyribose phosphate backbone) as constants compared to the methylation peak [53,54]. We chose 1160 cm^−1^ to make the I_1350_/I_1160_ ratio plot (Figure 5d), as it provided better comparisons between the synthetic samples in Figure 5 and cell line samples in Figure 6. We further constructed a linear regression graph with I_1350_ values only (Figure 5e); although Figure 5e’s graph had a better R^2^ value than Figure 5d’s did, we used Figure 5d values for our quantitative analysis because ratiometric quantification does not require an accurate measurement of DNA concentration in real samples. This approach has been reported several times previously [47,49]. We used the LINEST function for Figure 5d to determine the slope, intercept, and R^2^ values. We determined the limit of detection (LOD) using the 3σ/m formula and the limit of quantification (LOQ) using 10σ/m, where σ is the standard deviation and m is the slope of the calibration graph. We found that the LOD was 5% and the LOQ was 16% for the methylated DNA in the mixture of methylated and unmethylated DNA samples. Our detection limit was higher than PCR-SERS (~6%) [29] and nanotag-MBD (6.25%) [55]-based methods, and comparable to PGNA-SERS (~1%) [47] and single base extension-based methods (1%) [56].

As an additional study, to verify that the 1350 cm^−1^ was indeed due to methylation and not from changes in the general dsDNA structure, we checked SERS spectra of methylated and unmethylated DNAs with lower CpG densities that were randomly distributed throughout (Table 1). As indicated in Figure 5f, a methylation-specific peak at 1350 cm^−1^ was clearly shown in methylated DNAs with lower CpG densities.

### 2.4. Analysis of DNA Methylation in Colorectal Cancer Cell Genomic DNA

After successful quantification of methylation in synthetic DNA, we moved on to read the methylation status of colorectal cancer-specific cells’ genomic DNA. We chose SW48 and SW480 cell lines, as these are known to contain hypermethylated CpG sites [57,58]. SERS analysis of these two genomic DNA produced a red-shifted methylation-specific peak at 1358 cm^−1^ (Figure 6a,b). The red shift was due to environmental changes (e.g., other surrounding molecules in the cell samples). With a reducing amount of DNA, the peak intensity declined as well (Figure 6c). Moreover, correlating the intensity ratio (I_1358_/I_1160_) of 250 ng samples with the trendline in Figure 5d, we found that the global methylation level of SW48 cells was 55%, and of SW480 cells was 64% [16,57,59]. Linear regression graphs constructed with the DNA amount and SERS intensity ratio showed a positive correlation (Figure 6d,e).

Advantages of our method include the rich molecular fingerprint information of genomic DNA, no need for either prior bisulfite conversion and PCR, and direct and simple workflow. However, as there is no amplification step involved, detection sensitivity might be limited, which requires the design of a new nanostructure to improve detection sensitivity. Because our method is direct (label-free), direct DNA methylation analysis does not provide gene-specific methylation information. Therefore, comprehensive data analysis is normally required to quantify the methylation status.

## 3. Materials and Methods

### 3.1. General Information

Chloroauric acid (HAuCl4) and trisodium citrate tribasic dihydrate (C_6_H_5_Na_3_O_7_·2 H_2_O) were purchased from Sigma-Aldrich. MiliQ water (18.2 MΩ.cm, 25 °C) was used. Synthetic oligonucleotide and nuclease-free water (H_2_O) were procured from Integrated DNA Technologies (IDT) (Boronia, VIC, Australia). CpG methyltransferase (M.SssI) was purchased from New England Biolabs (NEB) (Notting Hill, VIC, Australia). Isolate II PCR and the Gel Kit used for DNA purification were purchased from Bioline (London, UK). A DNeasy Blood & Tissue Kit was collected from QIAGEN Pty Ltd (Clayton, VIC, Australia).

### 3.2. Spherical AuNPs Preparation

For preparing citrate-capped AuNPs, we first prepared 1% HAuCl_4_ in deionized water from a 1.5% stock solution. As the reducing agent, C_6_H_5_Na_3_O_7_·2 H_2_O 1% solution was prepared in deionized water. As we aimed to make 55 nm-sized particles specifically due to their higher signal enhancement, the HAuCl_4_:C_6_H_5_Na_3_O_7_·2 H_2_O ratio was carefully maintained. Firstly, in a fresh beaker, 49.5 mL MiliQ of water and 0.5 mL of 1% HAuCl_4_ were mixed. The solution was heated to boiling for 20 min with a magnetic rotation of 550 rpm. Once in boiling condition, 0.45 mL of 1% C_6_H_5_Na_3_O_7_·2 H_2_O was added and stirred for another 15 min. Next, the heat was turned off and the solution was stirred for a further 10 min. The beaker was kept at room temperature until it had cooled. It was then stored overnight at 4 °C. The next day experiments were performed.

### 3.3. Characterization of Nanoparticles

TEM imaging was performed using a Philips CM10 TEM (Eindhoven, The Netherlands). NTA was performed using a Nanosight NS300 instrument (Malvern Panalytica, Malvern, UK). Zeta potential (particle surface charge) was measured using a Zetasizer ZS instrument (Malvern Panalytica, Malvern, UK). Extinction spectra were measured with a JASCO V-760 UV-vis spectrophotometer (Kachioji, Tokyo, Japan). An IM-52 Raman microscopy instrument (Snowy Range Instruments, Laramie, WY, USA) was used for Raman and SERS measurements.

### 3.4. Methylated and Unmethylated DNA Preparation

Single-strand unmethylated DNA (sense strand) was heated to 95 °C and slowly cooled down to form a double-stranded duplex. To prepare methylated DNA in a microcentrifuge tube, 2 μL of 10× NEB buffer (provided with the M.SssI enzyme), 3 μL of double-stranded unmethylated DNA (100 μM), 5 μL of S-adenosyl methionine (3200 μM), 8 μL of H_2_O, and 2 μL of M.SssI (4 U/μL) were added. The reaction was incubated at 37 °C for 4 h. For the unmethylated control, in another tube, all reagents except the M.SssI were added. Reactions were purified using a DNA purification kit and eluted in H_2_O at a 50 ng/μL concentration. These sequences are described below.

### 3.5. Preparation of Cell Line DNA for SERS Analysis

SW48 and SW480 cell lines were cultured first. Using the DNeasy Blood & Tissue Kit, genomic DNA was extracted and diluted at a 50 ng/μL concentration. Following that, both synthetic and cell line DNA were analyzed by SERS with the same protocol in the next step.

### 3.6. Protocol for Detecting Label-Free SERS

First, 120 μL of AuNPs stock solution was placed in a microcentrifuge tube and centrifuged at 5400 rpm for 8 min. The suspension was removed, and 5 μL of (50 ng/μL) target DNA was mixed with AuNPs. Next, 55 μL 1× phosphate buffer saline was added to the tube. Finally, 5 μL 0.6 M NaCl (final concentration 0.05 mM) was added and directly transferred to the quartz cuvette for SERS analysis.

### 3.7. Setup for SERS Measurement

For solid sample analysis with the Raman spectrometer, samples were placed in front of the microscope and the lens was focused. Samples were excited at the 785 nm wavelength. The laser power was set at 70 mW with a 1 s acquisition time and three sets of readings.

For analysis in the liquid phase, samples were placed into a quartz cuvette. The same 785 nm excitation and 70 mW laser power were used, along with a 20 s acquisition time.

### 3.8. Denoising and Baseline Correction of Raman Spectra

For the processing of Raman spectra, we employed the Vancouver Raman Algorithm (VRA) to ensure accurate signal extraction and analysis [60,61]. The algorithm performs two key operations sequentially: denoising and baseline correction. First, a moving average filter is applied to raw spectra to reduce random noise and smooth the signal. This denoising step helped enhance the visibility of Raman peaks by averaging intensity values within a predefined window, minimizing high-frequency fluctuations in our data. Subsequently, baseline correction was carried out using the asymmetric least squares (ALS) smoothing method [60,61]. This approach iteratively fit a smooth baseline beneath the Raman signal by minimizing the effects of background noise and fluorescence. The ALS method utilized a smoothing parameter (λ) to control the smoothness of the baseline, and an asymmetry parameter (p) to ensure that the baseline predominantly remained beneath the signal peaks. Iterative adjustments were made to update the baseline and weight matrix, ensuring an accurate separation of the true Raman signal from background interference. Finally, corrected spectra, with noise reduced and baseline removed, were used for further qualitative and quantitative analysis. MATLAB 2024b software was used to process all spectra.

## 4. Conclusions

This article presents a label-free SERS method for the direct detection of CpG methylation in DNA. It utilizes the Raman enhancement potential of AuNPs and the fact that the C5 methyl group in 5mC has a unique signal. The step-change of this work is to control the assembly of DNA to nanoparticle hotspots with high ionic strength. The high specificity of SERS allowed clear differentiation between methylated and unmethylated states of DNA. Again, strong signal enhancement enabled the detection of DNA methylation as low as 5%. This method not only facilitated quantification from synthetic DNA, but also from genomic DNA of colorectal cancer cell lines. Overall, our study represents a straightforward amplification-free process for reading global DNA methylation with good selectivity and sensitivity.

## Figures and Tables

**Table 1:** Oligonucleotides used as unmethylated and methylated control DNA.

Oligonucleotide Name	Sequence
Unmethylated DNA	Sense: 5′-CGCGCGCGCGCGCGCGCGCGCGCGCGCGCGCGCGCGCGCGCGCGCGCGCGAAAAAAAAAA-3′
	Antisense: 3′-AAAAAAAAAAGCGCGCGCGCGCGCGCGCGCGCGCGCGCGCGCGCGCGCGCGCGCGCGCGC-5′
Methylated DNA	Sense: 5′-C_m_GC_m_GC_m_GC_m_GC_m_GC_m_GC_m_GC_m_GC_m_GC_m_GC_m_GC_m_GC_m_GC_m_GC_m_GC_m_GC_m_GC_m_GC_m_GC_m_GC_m_GC_m_GC_m_GC_m_GC_m_AAAAAAAAAA-3′
	Antisense: 3′-AAAAAAAAAAGC_m_GC_m_GC_m_GC_m_GC_m_GC_m_GC_m_GC_m_GC_m_GC_m_GC_m_GC_m_GC_m_GC_m_GC_m_GC_m_GC_m_GC_m_GC_m_GC_m_GC_m_GC_m_GC_m_GC_m_GC_m_-5′
Low CpG Unmethylated DNA	Sense: 5′-CGCTGACTCGACTGTGCAGTCGTGACTGCTCGAGACGTCGCATGTGCTAGAAAAAAAAAA-3′ Antisense: 3′-AAAAAAAAAAGCGACTGAGCTGACACGTCAGCACTGACGAGCTCTGCAGCGTACACGATC-5′
Low CpG methylated DNA	Sense: 5′-C_m_GCTGACTC_m_GACTGTGCAGTC_m_GTGACTGCTC_m_GAGAC_m_GTC_m_GCATGTGCTAGAAAAAAAAAA-3′ Antisense: 3′- AAAAAAAAAAGC_m_GACTGAGC_m_TGACACGTCAGC_m_ACTGACGAGC_m_TCTGC_m_AGC_m_GTACACGATC-5′

## Data Availability

Data that support the findings of this study are available from the corresponding authors upon reasonable request.
